# Spinodal Decomposition Method for Structuring Germanium–Carbon Li-Ion Battery Anodes

**DOI:** 10.1021/acsnano.2c12869

**Published:** 2023-04-17

**Authors:** Changshin Jo, Bo Wen, Hyebin Jeong, Sul Ki Park, Yeonguk Son, Michael De Volder

**Affiliations:** † Department of Engineering, University of Cambridge, 17 Charles Babbage Road, CB3 0FS Cambridge, United Kingdom; ‡ Graduate Institute of Ferrous & Energy Materials Technology (GIFT) and Department of Chemical Engineering, Pohang University of Science and Technology (POSTECH), Pohang 37673, Republic of Korea; § Cambridge Graphene Centre, Department of Engineering, University of Cambridge, 9 JJ Thomson Avenue, Cambridge CB3 0HE, United Kingdom; ∥ Department of Chemical Engineering, Changwon National University, Changwon 51140, Republic of Korea

**Keywords:** lithium ion batteries, anodes, germanium, carbon, spinodal decomposition

## Abstract

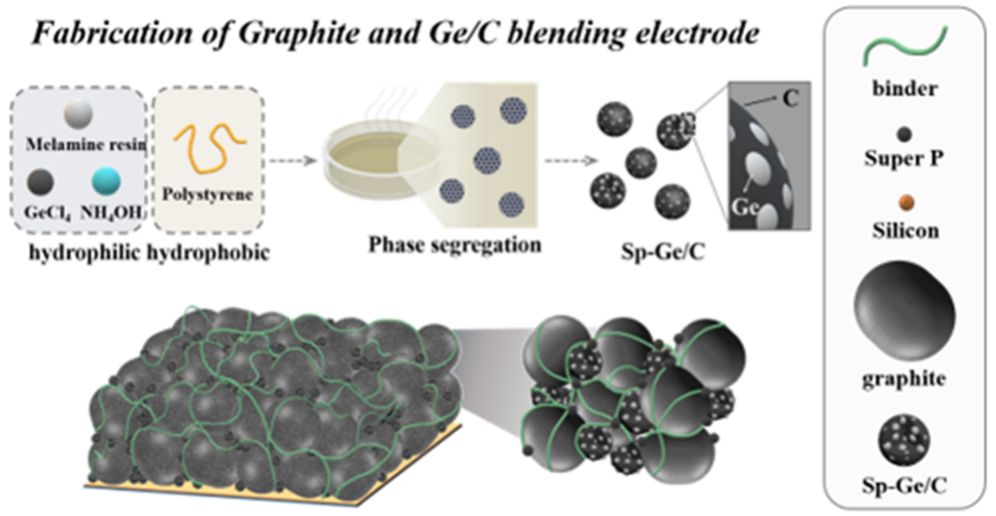

To increase the energy density of lithium-ion batteries (LIBs), high-capacity anodes which alloy with Li ions at a low voltage against Li/Li^+^ have been actively pursued. So far, Si has been studied the most extensively because of its high specific capacity and cost efficiency; however, Ge is an interesting alternative. While the theoretical specific capacity of Ge (1600 mAh g^–1^) is only half that of Si, its density is more than twice as high (Ge, 5.3 g cm^–3^; Si, 2.33 g cm^–3^), and therefore the charge stored per volume is better than that of Si. In addition, Ge has a 400 times higher ionic diffusivity and 4 orders of magnitude higher electronic conductivity compared to Si. However, similarly to Si, Ge needs to be structured in order to manage stresses induced during lithiation and many reports have achieved sufficient areal loadings to be commercially viable. In this work, spinodal decomposition is used to make secondary particles of about 2 μm in diameter that consist of a mixture of ∼30 nm Ge nanoparticles embedded in a carbon matrix. The secondary structure of these germanium–carbon particles allows for specific capacities of over 1100 mAh g^−1^ and a capacity retention of 91.8% after 100 cycles. Finally, high packing densities of ∼1.67 g cm^–3^ are achieved in blended electrodes by creating a bimodal size distribution with natural graphite.

## Introduction

Because of their high energy and power density, lithium ion batteries (LIBs) have become the preferred battery technology for applications ranging from portable electronic devices to electric vehicles. To maximize the energy density of LIB anodes, materials that can deliver highly reversible capacities at low voltage against Li are required. High-capacity alloying materials such as Si, Sn, and Ge have been studied extensively because their theoretical capacities are a multiple fold of that of standard commercial anode materials such as graphite and lithium titanate. However, during cycling, these materials undergo extreme volume changes, leading a range of different degradation mechanisms such as cracking of the material and excessive solid-electrolyte interphase (SEI) formation. To reduce mechanical degradation, nanosized particles are used, but unfortunately these suffer from low volumetric packing density and excessive SEI formation, leading to a low Coulombic efficiency (CE) and loss of Li inventory. These challenges are exacerbated when coating these materials in industrially relevant electrode formulations with high areal loading and small amounts of binder and conductive additive.

To alleviate some of these challenges, commercial anodes have been developed where a small percentage of graphite is replaced by high-capacity alloying materials (e.g., Si). In these so-called blended electrodes, the stability and conductivity of graphite mitigate some of the issues with the alloying materials discussed above. The alloying anode material, most commonly Si, is either coated on the graphite surface^1,2^ or more commonly physically mixed with graphite and ends up in pores between graphite particles.^3,4^ In some blended electrode studies, silicides (e.g., iron silicide) or oxides (SiO_*x*_, 0 ≤ *x* < 2) are used to reduce swelling and increase the conductivity of Si at the cost of a lower capacity.^5,6^ Highly impressive results have been achieved with these blended electrodes, which combine the advantages of intercalation and alloying reactions,^7−11^ resulting in this technology being commercialized successfully by several companies.^12^

Due to the relatively low cost and high specific capacity of Si, it has been studied more extensively than other alloying anodes, but compared to Sn and Ge, it has a disadvantage of lower electrical conductivity and larger volume expansion.^13^ In this work, we are comparing anodes with the same capacity made by blending either Si or Ge with graphite. The capacity of Ge (1600 mAh g^–1^) is only half that of Si, but it has a higher density (5.3 g cm^–3^; Si, 2.33 g cm^–3^), and therefore the charge stored per volume is similar to that of Si. In addition, it has a 400 times higher ionic diffusivity and 4 orders of magnitude higher electronic conductivity compared to Si.^14−17^

Previous studies have shown promising results using nanostructured Ge or its composite materials; however, this was obtained under a low mass loading condition. Under realistic loading levels needed for commercial applications, Ge anodes tend to fail because of excessive swelling leading to poor structural stability of the electrode and excessive SEI formation, in particular when using nanosized active particles. In order to reduce SEI formation,^18−22^ our strategy is to create secondary particles where Ge nanoparticles are embedded in a micrometer-sized secondary particle. These micrometer-sized aggregates reduce surface-driven degradation mechanisms and lead to good packing density when coating the electrode. In order to cement the Ge nanoparticles together, a carbon matrix is selected, as it is both electrically and ionically conductive. However, creating germanium–carbon (Ge/C) secondary particles with a micrometer-sized diameter and good size distribution is challenging. In this work, both objectives were achieved by using spinodal decomposition to template the active materials.^23−25^ In our case, we start by dispersing Ge and carbon precursors with polystyrene in THF, which results in a homogeneous dilute dispersion. We then evaporate THF, and at a certain threshold concentration, this homogeneous mixture spontaneously separates into two phases, where one contains the Ge and carbon precursors and the other polystyrene, which is subsequently washed off. This process does not require expensive apparatus or surfactants and results in individualized particles with a tight size distribution. Further, there have been many reports on making nanosized composites of carbon with alloying materials (mainly Si), but only a few papers have presented controlled micrometer-sized composites (and especially not Ge), which we believe is important to reduce the surface area and achieve good packing densities.^6,26^ The latter can be further improved by designing the germanium and carbon particle size to be smaller than that of graphite particles, such that a bimodal particle distribution is obtained where the Ge/C particles can fit in the crevices between graphite. Combining the above strategies, this paper demonstrates blends of Ge/C secondary particles and graphite that achieve a better cycle life, 443 mAh g^–1^ (92% retention after 60 cycles at 200 mA g^–1^) with 1.67 g cm^–3^ electrode density, and lower electrode swelling (40.9%) compared to control experiments using a blend of commercial Si nanoparticles (NPs) and graphite (398 mAh g^–1^ with 1.61 g cm^–3^ electrode density and 84% swelling).

## Results and Discussion

### Preparation and Characterization of Spherical Germanium and Carbon Composite

**Figure 1:**
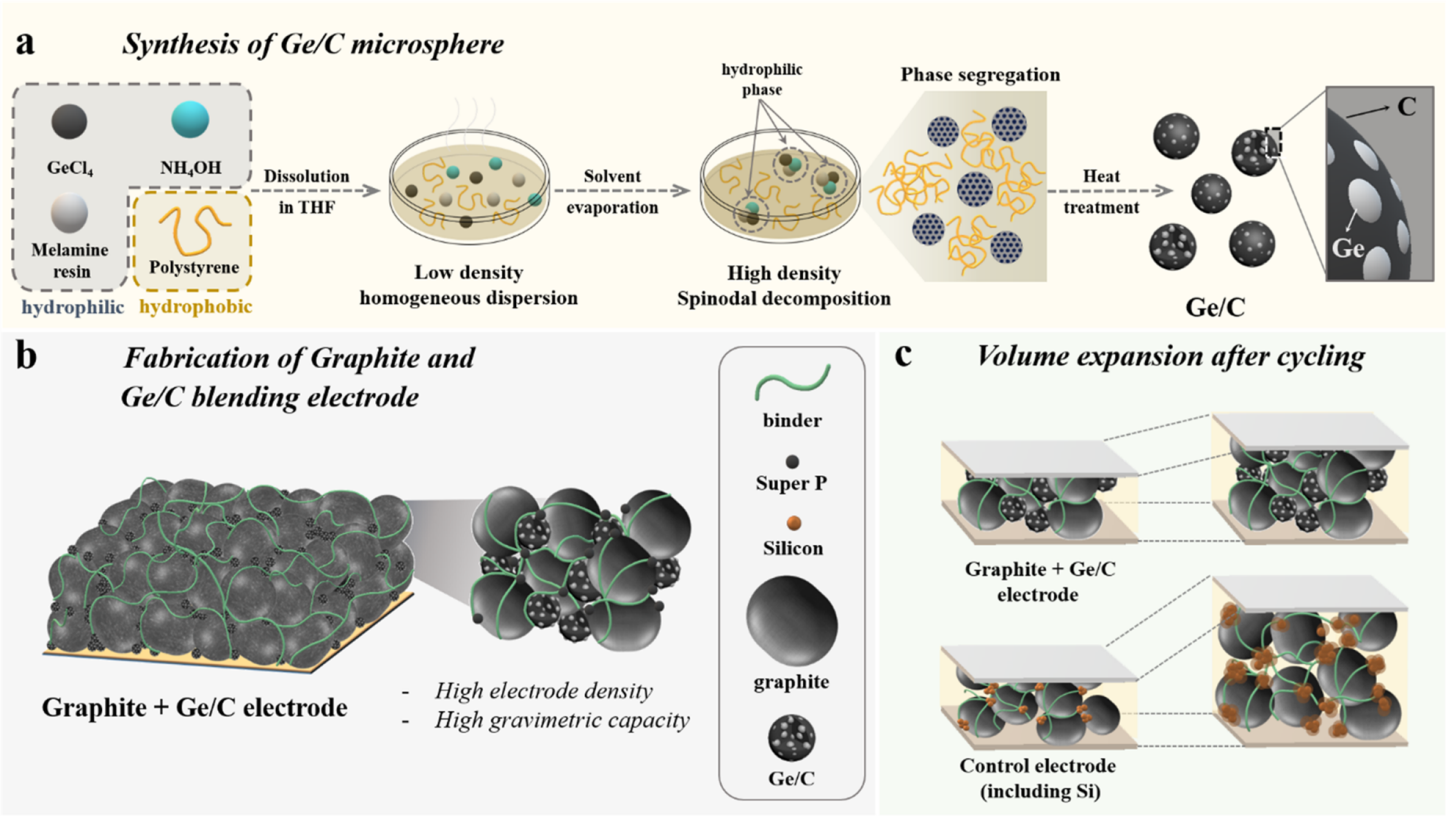
Schematic illustrations of (a) synthesis process of Sp-Ge/C microparticles using spinodal decomposition, (b) blended electrodes (commercial graphite, Sp-Ge/C, carbon additive, and polymeric binder), and (c) volume expansion of Graphite+Sp-Ge/C and Graphite+Si NPs electrodes after cycling.

At the core of our process, we leverage spinodal decomposition to create secondary germanium microparticles embedded in a carbon matrix (Sp-Ge/C). Spinodal decomposition is a spontaneous phase separation process occurring in certain mixtures: in this case, in solutions with different miscibility depending on the concetration.^25,27,28^ By changing the solution concentration by evaporation, two or more phases can be created in a short time frame. Researchers have applied this separation technique to design complicated nano/macro structures previously^23,24,29,30^ but have not applied this process to create advanced alloying anodes. The process developed in this study is illustrated in Figure 1a. First, Ge and C (GeCl_4_ and melamine resin, respectively) precursors are dissolved in an aqueous NH_4_OH solution (sol–gel catalyst). This aqueous phase is not miscible with a hydrophobic homopolystyrene (hPS) phase. We used THF as a cosolvent that can dissolve both precursors and homopolymer to codisperse all the materials discussed above. We then evaporate THF, which makes the remaining mixture unstable and initiates a phase separation of micrometer-sized particles consisting of Ge/C/NH_4_OH in a hPS matrix. Here, the volume of the hydrophilic phase was chosen to be smaller than that of hPS such that spherical particles of the precursors are formed that are surrounded by hPS. Within the Ge/C/NH_4_OH phase, aqueous NH_4_OH solutions can control the hydrolysis and condensation reactions of Ge precursors,^31−33^ which forms different nanometer-sized GeO_*x*_ particles and controls the miscibility to the hPS phase.^23^

After the phase segregation and drying processes are completed, the as-made sample is collected and washed with THF to remove hPS. Next, the particles are annealed at 700 °C in a He–H_2_ gas atmosphere (He for 50 min, 10% H_2_ in He for 10 min) to reduce GeO_*x*_ to Ge and to pyrolyze the melamine resin. We also attempted to further decrease the surface area and improve the stability of Sp-Ge/C in a battery anode application by coal tar pitch coating.^11^ In this process, Sp-Ge/C is mixed with coal tar pitch using THF, followed by drying and heat treatment at 700 °C under He (Sp-Ge/C-Pitch).

**Figure 2:**
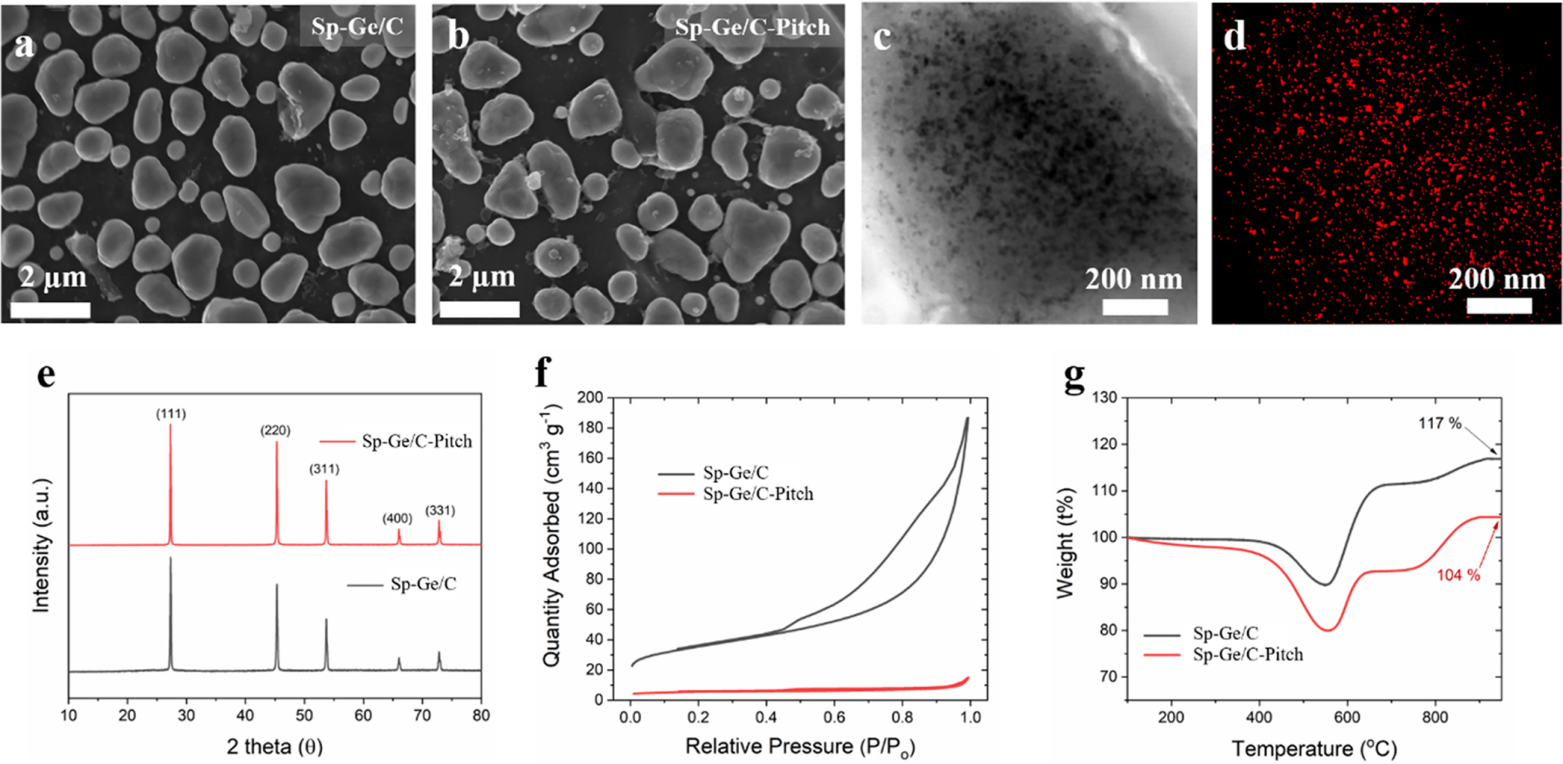
Scanning electron microscopy (SEM) images of (a) Sp-Ge/C and (b) Sp-Ge/C-Pitch. (c) Transmission electron microscopy (TEM) and (d) Energy-dispersive X-ray spectroscopy images of Sp-Ge/C-Pitch. (e) X-ray diffraction patterns, (f) nitrogen physisorption, and (g) thermogravimetry curve data of Sp-Ge/C and Sp-Ge/C-Pitch samples.

The resulting sample has 1–2 μm sized particles containing ∼30 nm Ge particles in an amorphous carbon matrix (see Figure 2). The size of these Sp-Ge/C particles was selected to be smaller than that of the commercial graphite particles (∼17 um) used in this work, such that a bimodal particle distribution is obtained, where it is known that the small particles fit in the gaps that naturally occur between graphite particles (see Figure 1b). This leads to higher electrode densities and fewer issues with particle swelling. Compared to commercial Si NPs and graphite blended electrodes, mostly investigated as high-energy-density anodes, the Sp-Ge/C and graphite blended electrode exhibited less volume expansion after cycling (see below). Further, the method described above essentially packs Ge nanoparticles into secondary aggregates, which is a popular method in industry to improve the processability of materials.

To control the phase segregation, we tested several base and acid solutions including HCl, HNO_3_, and NH_4_OH solution as hydrophilic sol–gel catalysts and found that NH_4_OH was the only one resulting in well-defined rounded microparticles (Figure S1). Scanning electron microscopy (SEM) images of Sp-Ge/C in Figure 2 show rounded isolated particles that are 1–2 μm in diameter. The existence of carbon can be confirmed by Raman spectroscopy (Figure S2). Both disordered (1335 cm^–1^) and graphitic (1580 cm^–1^) peaks were measured, which indicates the sample contains amorphous carbon. After the coal tar pitch coating process, the particles retain their shape, as shown in Figure 2b. In addition, Figure 2c,d shows a good distribution of Ge particles in the carbon matrix, observed by transmission electron microscopy (TEM) and energy-dispersive X-ray spectroscopy (EDX). In the high-resolution TEM image (Figure S3a), ∼30 nm Ge NPs are embedded inside an amorphous carbon matrix. The spacings between lattice fringes are 3.2 Å, corresponding to the (111) lattice plane of the cubic Ge phase (Figure S3b). Further, a line-scan analysis shows that the elemental Ge signal is centered at the core of the Sp-Ge/C-Pitch particle (Figure S4).

Heat treatment in inert gas (He) generates the GeO_2_ phase (JCPDS no. 85-0473, Figure S5), converted from GeO_*x*_ sol, and reductive gas (H_2_) reduces GeO_2_ into Ge particles (Sp-Ge/C). X-ray diffraction (XRD) patterns of both Sp-Ge/C and Sp-Ge/C-Pitch samples were assigned as cubic phase Ge (JCPDS no. 04-0545, Figure 2e). The BET (Brunauer–Emmett–Teller) surface areas of Sp-Ge/C and Sp-Ge/C-Pitch are 120 and 17 m^2^ g^–1^, respectively (Figure 2f). Coal tar pitch derived carbon is expected to stabilize the active materials during battery operation by alleviating the volume change and stabilizing the surface-originated side reaction.^34^ Thermogravimetry analysis (TGA) was used to measure the mass ratio of Ge and carbon (Figure 2g). After TGA analysis in air, Sp-Ge/C and Sp-Ge/C-Pitch showed 117% and 104% mass, respectively, compared to the initial conditions. Considering that the oxidation of Ge into GeO_2_ increases the mass up to 144% and assuming all the Ge was reduced at the start of the experiment, the Ge content in Sp-Ge/C and Sp-Ge/C-Pitch are assumed to be 81% and 72%, respectively.

### Electrochemical Performance of Germanium and Carbon Anodes

**Figure 3:**
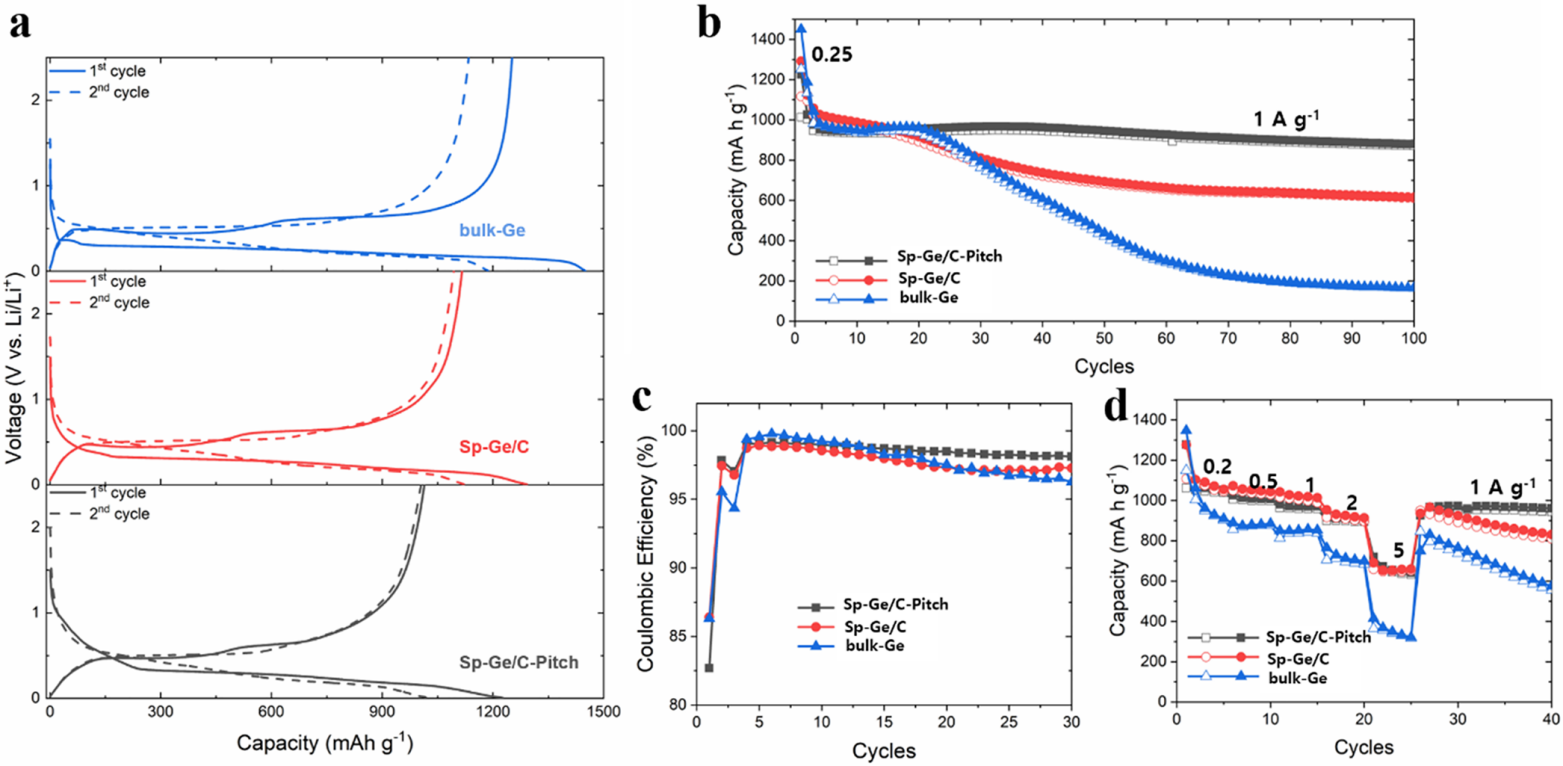
(a) Galvanostatic charge/discharge profiles, (b) cycling performance, (c) Coulombic efficiency, and (d) rate performance data of Sp-Ge/C, Sp-Ge/C-Pitch, and bulk-Ge electrodes.

To evaluate the electrochemical performance of Sp-Ge/C-Pitch compared to other anode formulations, galvanostatic charge/discharge tests were first performed in a half-cell configuration (Figure 3). Active materials were mixed with a carboxymethyl cellulose (CMC)-styrenebutadiene rubber (SBR) binder and carbon additive (Super P) in DI water (8:1:1 mass ratio). A bulk-Ge-powder-based electrode was prepared as a control sample. 1.3 M LiPF_6_ in EC/DEC with 10 wt % FEC was used as an electrolyte (see Methods).

Figure 3a shows charge/discharge profiles of bulk-Ge (blue), Sp-Ge/C (red), and Sp-Ge/C-Pitch (black) electrodes under 0.25 A g^–1^ current density. In the first cycle, all electrodes have similar charge/discharge profiles showing two lithiation plateaus (at 0.35 and 0.25 V vs Li/Li^+^) and delithiation plateaus (at 0.5 and 0.65 V vs Li/Li^+^). Sp-Ge/C and Sp-Ge/C-Pitch electrodes delivered reversible capacities of 1117 and 1013 mAh g^–1^, respectively. On the other hand, bulk-Ge had a 1252 mAh g^–1^ capacity. This is because the carbon phase offers less capacity contribution than Ge. The Sp-Ge/C-Pitch formulation achieves a capacity retention of 91.8% after 100 cycles at a current density of 1 A g^–1^ (Figure 3b). On the other hand, Sp-Ge/C and bulk-Ge electrodes retained 59.6 and 16.7% of their capacities, respectively. In addition, in the charge/discharge profiles and d*Q*/d*V* plots, Sp-Ge/C-Pitch samples exhibited a highly reversible and stable charge/discharge process (more charge/discharge and d*Q*/d*V* curves are provided in Figures S6 and S7). Sp-Ge/C-Pitch electrodes maintained 68.1% of their capacity (645 mAh g^–1^) after 300 cycles; in contrast, Sp-Ge/C cells failed entirely after 215 cycles (Figure S8). The improved stability of the Sp-Ge/C-Pitch electrode is probably due to the introduction of both the carbon matrix from the melamine precursor and the pitch-derived carbon shell on the particles.^35−37^

The Sp-Ge/C-Pitch electrode has a slightly lower initial CE (ICE) value of 82.7% (Sp-Ge/C, 86.4%, bulk-Ge, 86.3%). However, this is still very high considering that we are using nanosized alloying materials and the CE values of Sp-Ge/C-Pitch are higher than those of other electrodes in subsequent cycling (Figure 3c). CE values of Sp-Ge/C-Pitch electrodes were stable over 300 cycles. However, Sp-Ge/C and bulk-Ge electrodes showed decreasing CE values for a few tens of cycles and were slowly stabilized compare to CE values of Sp-Ge/C-Pitch (Figure S9). In the rate performance test (Figure 3d), the Sp-Ge/C-Pitch and Sp-Ge/C electrodes showed similar capacity retention: Sp-Ge/C-Pitch and Sp-Ge/C electrodes retain 65.8% (699 mAh g^–1^) and 59.4% (659 mAh g^–1^) capacities at 5 A g^–1^. For comparison, bulk-Ge electrodes retain only 31.8% (365 mAh g^–1^) at 5 A g^–1^.

After testing the proposed Sp-Ge/C-Pitch microparticles, we mixed them with commercial natural graphite (NG) powder in a 1:4 mass ratio to form blended electrodes as discussed above. The active materials were mixed with conductive carbon (Super P) and binder (CMC-SBR) in a 91:3:6 mass ratio. After coating, the electrodes were calendared to increase the electrode density and decrease contact resistance. The blended electrodes reach up to 1.75 g cm^–3^ densities thanks to the high gravimetric density of Ge as well as the Sp-Ge/C-Pitch secondary microparticle structure. Considering that commercial graphite electrodes typically have electrode densities up to 1.5–1.6 g cm^–3^ and this value decreases with increasing Si NP content, the reported electrodes offer an interesting option to increase electrode density. For a half-cell test, we prepared Sp-Ge/C-Pitch + graphite electrodes, which were calendared to have a density of 1.67 g cm^–3^. For the control experiment, we prepared Si NPs + graphite blended electrodes using commercial Si NPs (50 nm, 6 wt %) with a comparable electrode density of 1.61 g cm^–3^. The areal mass loading of active materials in each sample was about 6.0–6.3 mg cm^–2^.

**Figure 4:**
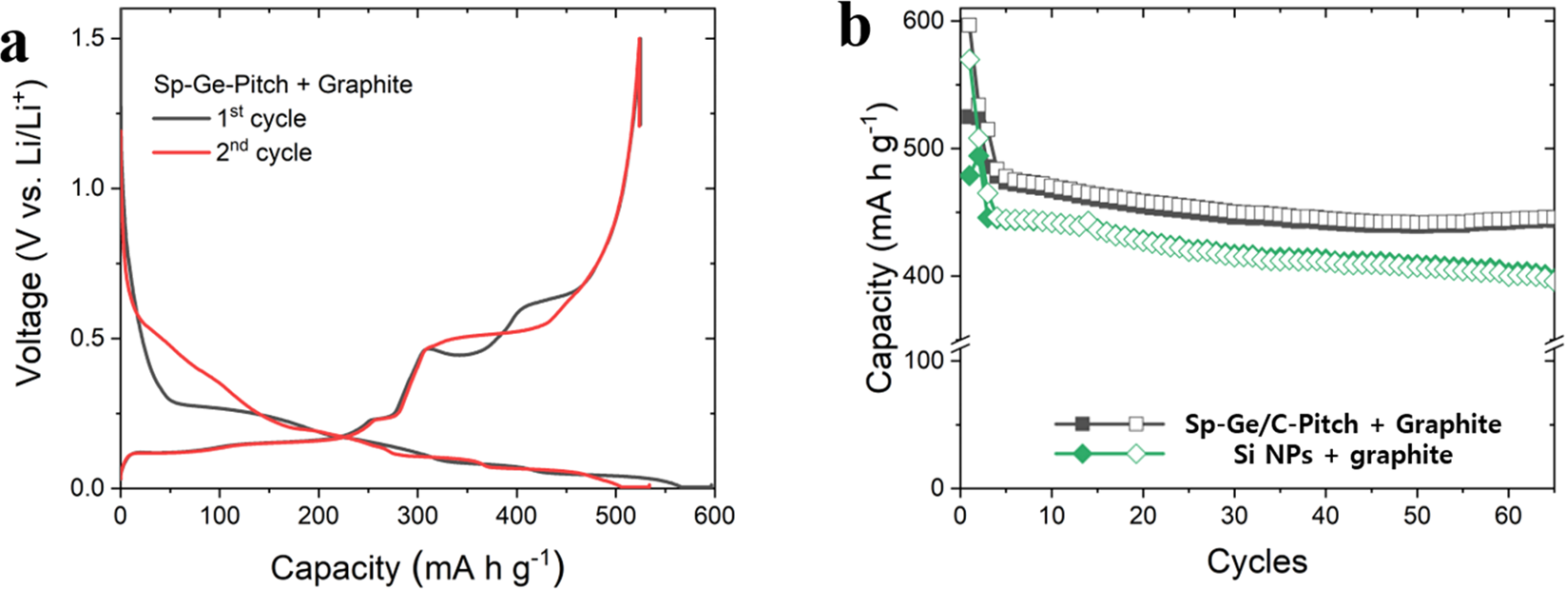
(a) Charge/discharge profile of Sp-Ge/C-Pitch + graphite electrode and (b) cycling performance of Sp-Ge/C-Pitch + graphite electrode and Si NPs + graphite electrode.

Figure 4 a shows the charge/discharge profile of the Sp-Ge/C-Pitch + graphite electrode at a current density of 50 mA g^–1^. The initial lithiation/delithiation capacities were 597 and 525 mAh g^–1^, respectively (ICE: 87.9%). At the second cycle, the electrode showed a different delithiation curve but delivered the same capacity (CE: 98.1%). On the other hand, the Si NPs + graphite electrode showed 84.0% (570 and 479 mAh g^–1^) and 97.2% (508 and 494 mAh g^–1^) CE values at the first and second cycles, respectively. After two formation cycles, at a current density of 200 mA g^–1^, the Sp-Ge/C-Pitch + graphite electrode exhibited a delithiation capacity of 486 mAh g^–1^, while the capacity of the Si NPs + graphite electrode decreased to 446 mAh g^–1^ (Figure 4b and Figure S10). After 60 cycles (at 200 mA g^–1^), the Sp-Ge/C-Pitch + graphite electrode maintained a capacity of 443 mAh g^–1^. However, in the Si blended graphite electrode, the reversible capacity decreased to 398 mAh g^–1^. This indicates that in our experiments Sp-Ge/C-Pitch secondary particles have a better cycling performance under high electrode density and capacity. In addition, in the rate performance test (Figure S11), the Sp-Ge/C-Pitch blended electrode showed higher capacity values at 500 and 1000 mA g^–1^ current densities, possibly due to the intrinsically higher ionic and electronic conductivities of Ge compared to those of Si.

**Figure 5:**
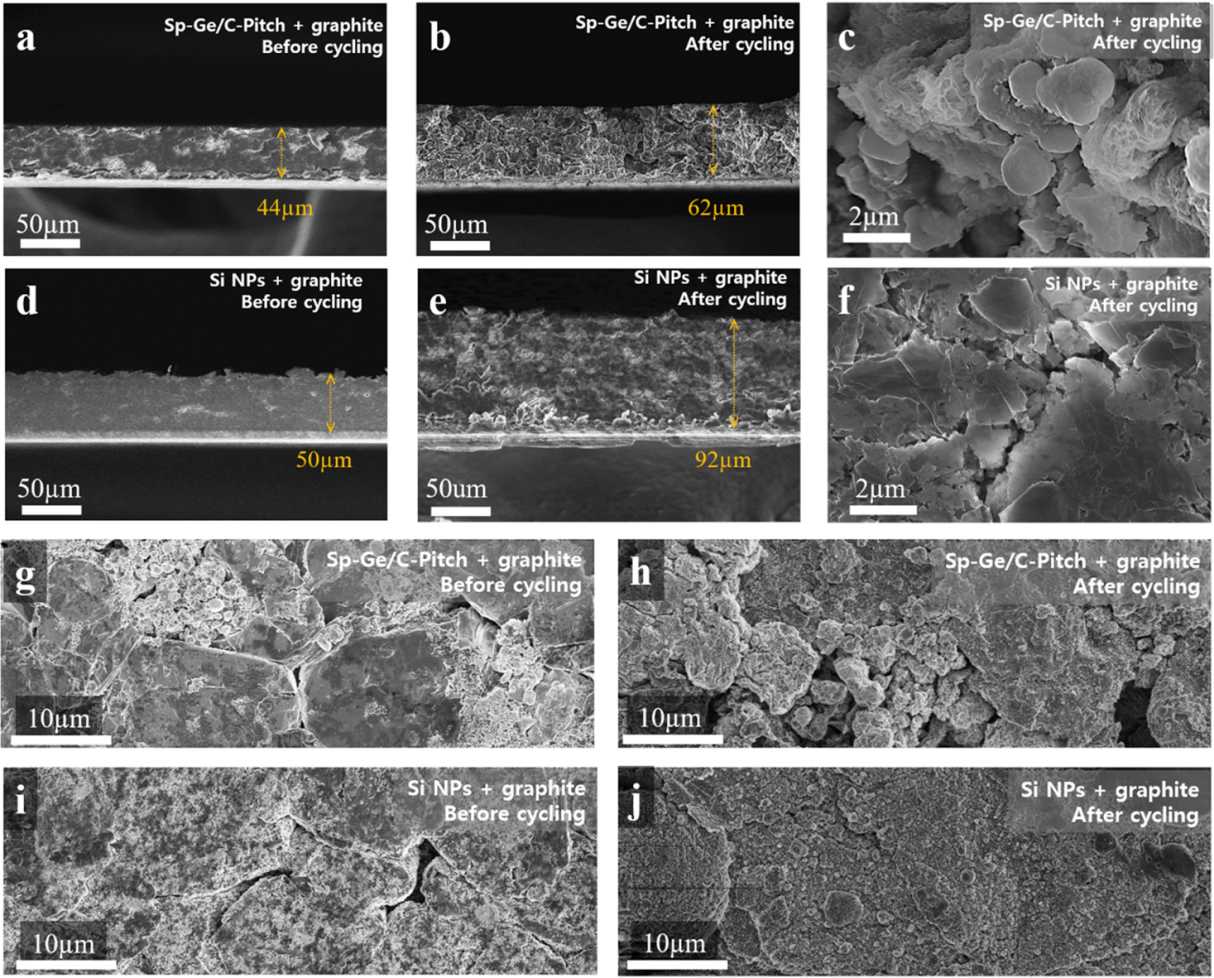
Cross-section SEM images of the Sp-Ge/C-Pitch + graphite electrode (a and g) before and (b, c, and h) after cycling and the Si NPs + graphite electrode (d and i) before and (e, f, and j) after cycling.

In Figure 5, we show cross-section images of Sp-Ge/C-Pitch and Si NP blended electrodes with the same electrode density (∼1.6 g cm^–3^) before and after cycling (cycled electrodes were washed in DEC). After 50 cycles at 200 mA g^–1^, Sp-Ge/C-Pitch + graphite electrodes show a volume expansion of 41% (44 to 62 μm) whereas the Si NPs + graphite electrode expanded by 84% (50 to 92 μm) and showed a thick SEI layer (Figure 5f,j and Figure S12). This indicates that our Ge-carbon secondary particles maintain their structural integrity, which is also apparent from their spherical shape and particle sizes in Figure 5c after cycling.

Finally, our blended electrodes were tested in a full cell configuration,^5,6,38^ against commercial LiCoO_2_ (LCO) cathodes (Figure S13). After a formation cycle at 0.1 C, the cells were cycled at a 0.5 C. The anode to cathode ratio was adjusted to ∼1.1. At the formation cycle, the Si NPs + graphite//LCO cell showed an ICE value of 84.1% (discharge capacity: 148 mAh g_LCO_^–1^), whereas the Sp-Ge/C-Pitch + graphite//LCO cell yielded an ICE value of 88.8% (discharge capacity: 154 mAh g_LCO_^–1^). In the charge/discharge curve (Figure S13a), the integrated area is proportional to the energy density of the full cell. In the Sp-Ge/C-Pitch + graphite//LCO cell, the ratio of discharge area to charge area is 81.3%, which is higher than that of the Si NPs + graphite//LCO cell (77.8%), indicating better energy efficiency in germanium and carbon blended anode cell. Finally, the Ge-based full cell exhibited better capacity values for 50 cycles (Figure S13b).

## Conclusions

In summary, Ge is an interesting battery alloying material because of its high energy density and conductivity. However, due to the swelling issue, nanosized Ge particles are needed to manage mechanical stresses in these materials during cycling. The latter results in a poor packing density of the electrodes as well as excessive SEI formation. Here we show a method using spinodal decomposition to create micrometer-scale secondary particles consisting of a mixture of Ge and carbon without the need for any expensive apparatus. This process results in a low surface area and allows for a high electrode density. The diameter of these secondary particles is optimized to fit in the natural gaps between graphite particles such that high density and stable blended electrodes can be fabricated. Overall, these electrodes resulted in higher rate performance and cycling stability than unstructured control materials.

## Methods

### Materials

Germanium(IV) chloride, tetrahydrofuran (THF), ammonia solution (25%), polystyrene (350 kg mol^–1^), melamine, and germanium particles were purchased from Sigma-Aldrich.

### Synthesis of Germanium and Carbon Composite

A 3 g portion of polystyrene was dissolved in 30 mL of THF to make a clear polymeric solution. to this solution was slowly added GeCl_4_/THF solution (0.6 mL of GeCl_4_ in 5 mL of THF). After mixing, 0.5 mL of ammonia solution and 0.2 g of melamine were added to the solution. After stirring for 1 h, the mixed solution was cast onto a glass dish placed on a hot plate at 40 °C. THF and H_2_O (from ammonia solution) were slowly evaporated for several hours. After solvent evaporation, the sample was further annealed at 100 °C overnight. After formation of the structure (Ge and carbon phase), the sample was soaked in THF to dissolve the polystyrene homopolymer again. After centrifugal separation and washing, the collected powder was heat-treated at 700 °C in a He–H_2_ gas atmosphere (He for 50 min, 10% H_2_ in He for 10 min, Sp-Ge/C). To coat Pitch-derived carbon, the spherical Ge/carbon composite particles were dispersed in Pitch/THF solution (the Ge/C particle to Pitch mass ratio was 4:1). After drying THF at 50 °C, the powder was heat-treated at 700 °C in He for 1 h (Sp-Ge/C-Pitch).

### Material Characterization

Morphologies of all samples were observed by scanning electron microscopy (SEM, a Leo Variable Pressure instrument) and transmission electron microscopy (TEM, TEM-1011, Jeol LTD). X-ray diffraction (XRD) analysis was performed using a Bruker D8 Advance (Cu Kα radiation, 4° min^–1^ scan). Nitrogen physisorption was measured by using a Micromeritics TriFlex adsorption analyzer. Thermogravimetric analysis (TGA) was performed using a PerkinElmer Pyris1 instrument under an air atmosphere (3 °C min^–1^). Raman spectroscopy was performed by using a Bruker Ram II-Senerra instrument.

### Electrode Preparation

The Sp-Ge/C-Pitch, Sp-Ge/C, and bulk-Ge samples were mixed with Super P carbon, carboxymethyl cellulose (CMC, MTI Corp.) and styrene–butadiene rubber (SBR, MTI Corp.) with a mass ratio of 8:1:0.5:0.5 in DI water. For the blended electrode, active materials, Sp-Ge/C-Pitch and graphite mixture (1:4 mass ratio) or Si NPs (∼50 nm) and graphite mixture (6:94 ratio), were separately mixed with Super P and binder (CMC-SBR) in a 91:3:6 mass ratio. The mixed slurry was cast on copper foil and dried in a 100 °C vacuum oven for 12 h. After the drying process, the electrodes were pressed to increase the electrode density. For the LiCoO_2_ (LCO) cathode, LCO was mixed with Super P and polyvinylidene fluoride (PVDF) with a mass ratio of 94:3:3 using *N*-methyl-2-pyrrolidone (NMP), followed by drying and calendaring. To fabricate a full cell, blended electrodes were assembled with LCO electrodes (the anode to cathode ratio was adjusted to ∼1.1).

### Electrochemical Characterization

CR2032-type coin cells were assembled inside the glovebox. Metallic Li foil was used as a counter/reference electrode. For all cells, 1.3 M LiPF_6_ in ethylene carbonate/diethyl carbonate (EC/DEC, Sigma-Aldrich) with 10 wt % fluoroethylene carbonate (FEC, Insight Biotechnology, Ltd.) was used as an electrolyte. All coin cells were tested at 25 °C under a constant current mode (CC mode for Ge electrodes) and constant current–constant voltage mode (CC-CV mode for blended electrodes) with a potential range of 0.005–1.5 V (or to 2.5 V vs Li/Li^+^). A LAND cycler (Wuhan Land Electronic Co., Ltd.) and Biologic VMP3 instrument were used to collect data. For ex situ SEM experiments, coin cells were disassembled in a glovebox and the electrodes were washed with DEC.

## Supporting Information Available

SEM/TEM images of GE/C composite, Raman of spherical GeO_2_/C composite, XRD of spherical GeO_2_/C composite, and charge/discharge, dQ/dV plots, rate performance data, SEM, and full-cell data of Sp-Ge-Pitch + graphite and Si NPs + graphite electrodes (PDF)
