# Supplementary material for: Spinodal Decomposition Method for Structuring Germanium–Carbon Li-Ion Battery Anodes
Source: ACS Nano. 2023 Apr 17;17(9):8403–10. doi: 10.1021/acsnano.2c12869 (PMC10173680; doi:10.1021/acsnano.2c12869)
Supplement: Supplementary file 1 — nn2c12869_si_001.pdf [file nn2c12869_si_001.pdf]

A Spinodal Decomposition Method for Structuring Germanium-Carbon Li-Ion Battery Anodes

Changshin Jo[a,b,‡,*], Bo Wen[a,c, ‡], Hyebin Jeong[b], Sul Ki Park[a], Yeonguk Son[a,d], Michael De Volder[a,*]

[a] Department of Engineering, University of Cambridge, 17 Charles Babbage Road, CB3 0FS Cambridge, United Kingdom

[b] Graduate Institute of Ferrous & Energy Materials Technology (GIFT) and Department of Chemical Engineering, Pohang University of Science and Technology (POSTECH), Pohang 37673, Republic of Korea

[c] Cambridge Graphene Centre, Department of Engineering, University of Cambridge, 9 JJ Thomson Avenue, Cambridge CB3 0HE, United Kingdom

[d] Department of Chemical Engineering, Changwon National University, Changwon, 51140, Republic of Korea

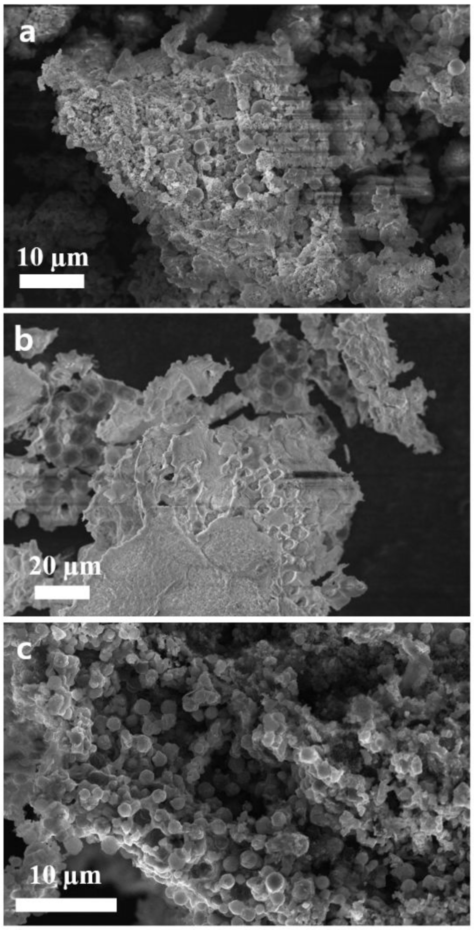

Figure S1. Scanning electron microscope images of Ge/C composite synthesized under different acid/base catalyst conditions; (a) HCl (37%), (b) HNO_3 (70%), and (c) Ammonia solution (25%).

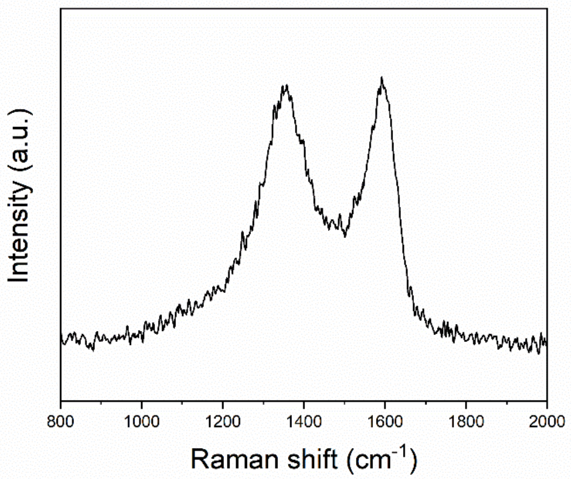

Figure S2. Raman spectroscopy of Sp-Ge/C sample.

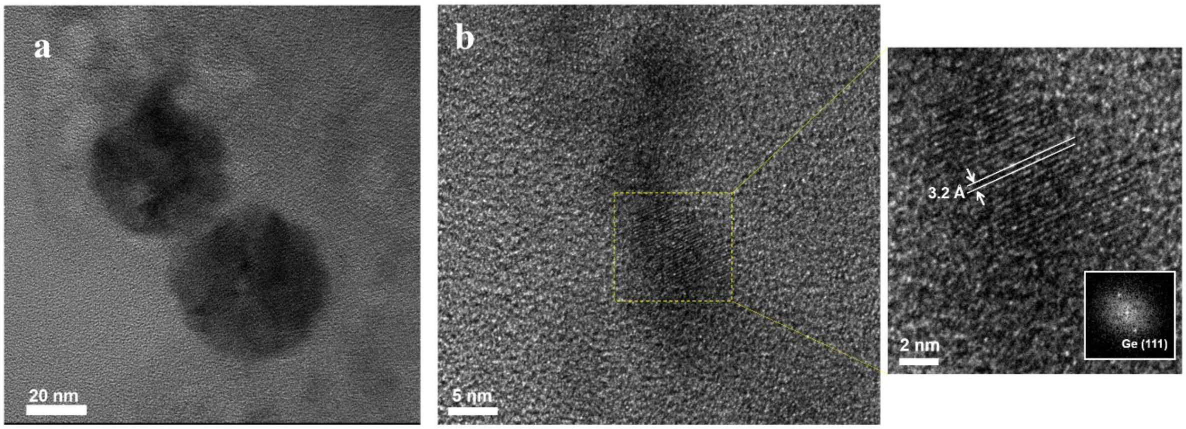

Figure S3. High-resolution transmission electron microscope images of Sp-Ge/C-Pitch

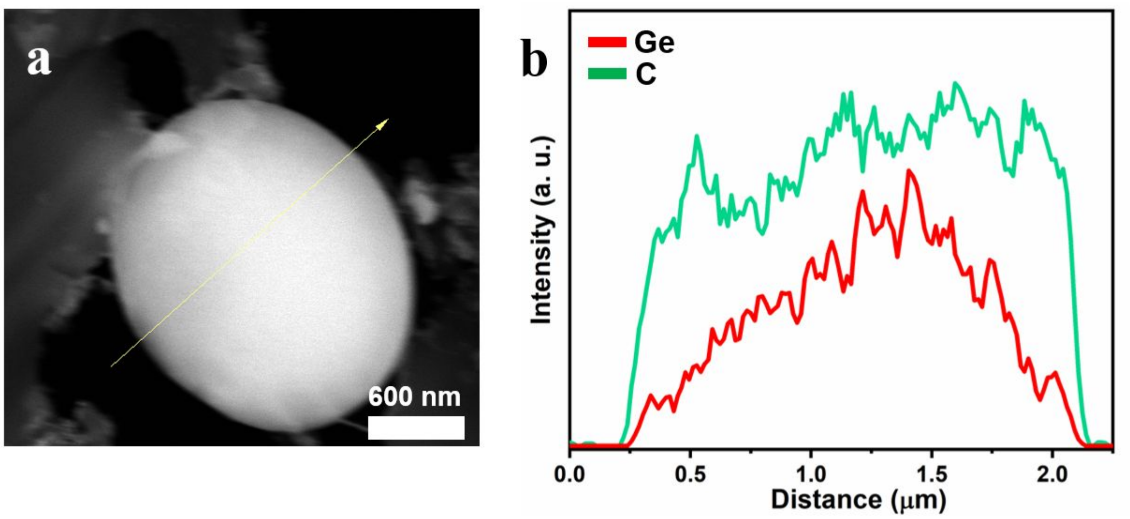

Figure S4. Line-scan analysis of transmission electron microscope image of Sp-Ge/C-Pitch sample.

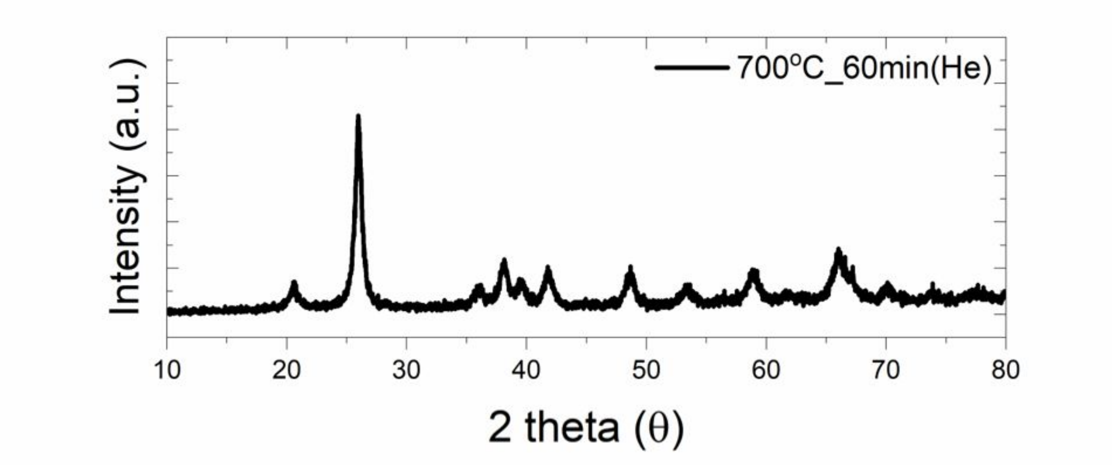

Figure S5. X-ray diffraction pattern of spherical GeO_2/C composite (hexagonal GeO_2, JCPDS no.: 85-0473).

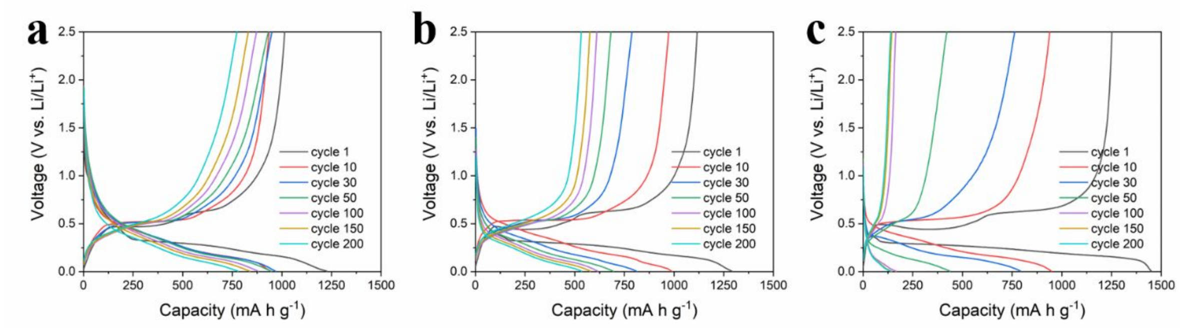

Figure S6. Charge/discharge curves of a) Sp-Ge/C-Pitch, b) Sp-Ge/C, and c) bulk-Ge electrodes for 200 cycles, obtained under 0.25 A g^{-1} (cycle 1) and 1 A g^{-1} (10th to 200th cycle) current densities.

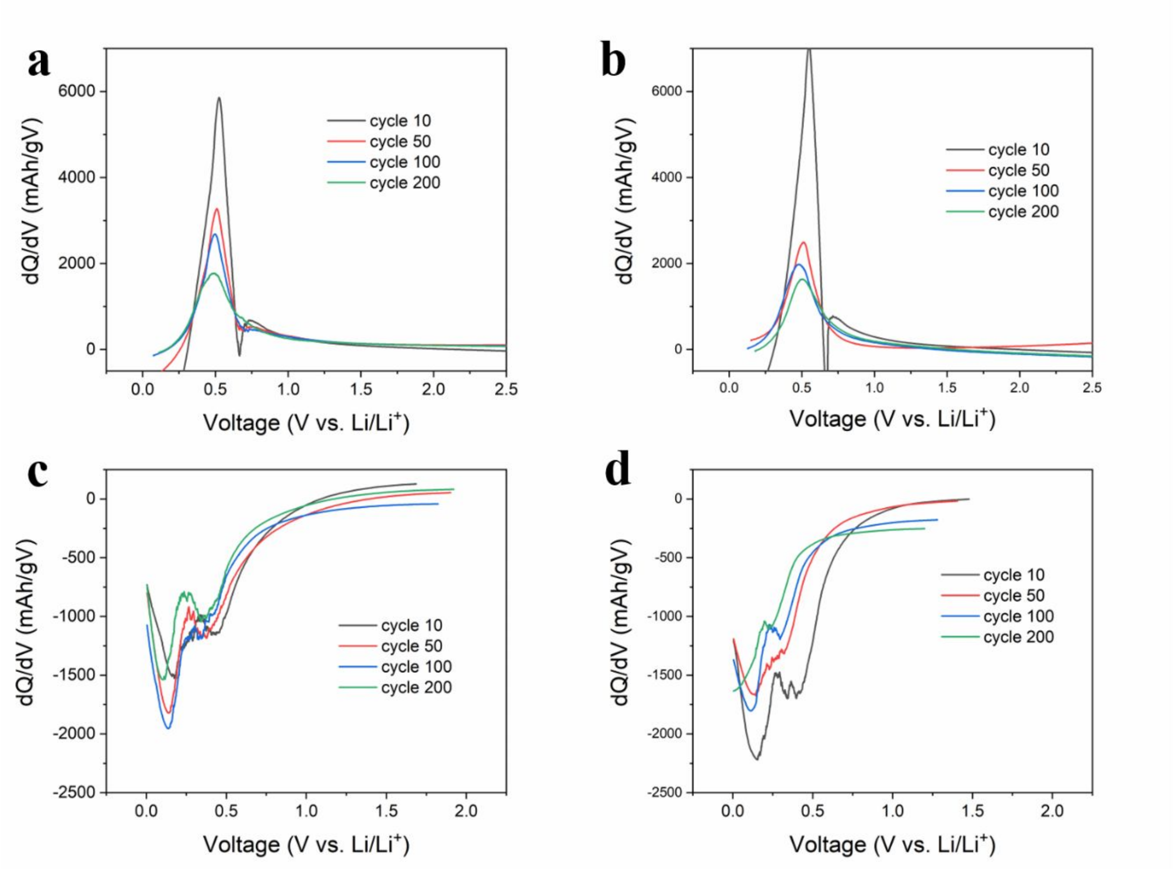

Figure S7. dQ/dV plots (10~200th cycles) for (a and b) delithiation and (c and d) lithiation processes measured under 1 A g^{-1} current density; (a and c) Sp-Ge/C-Pitch electrode and (b and d) Sp-Ge/C electrode, respectively.

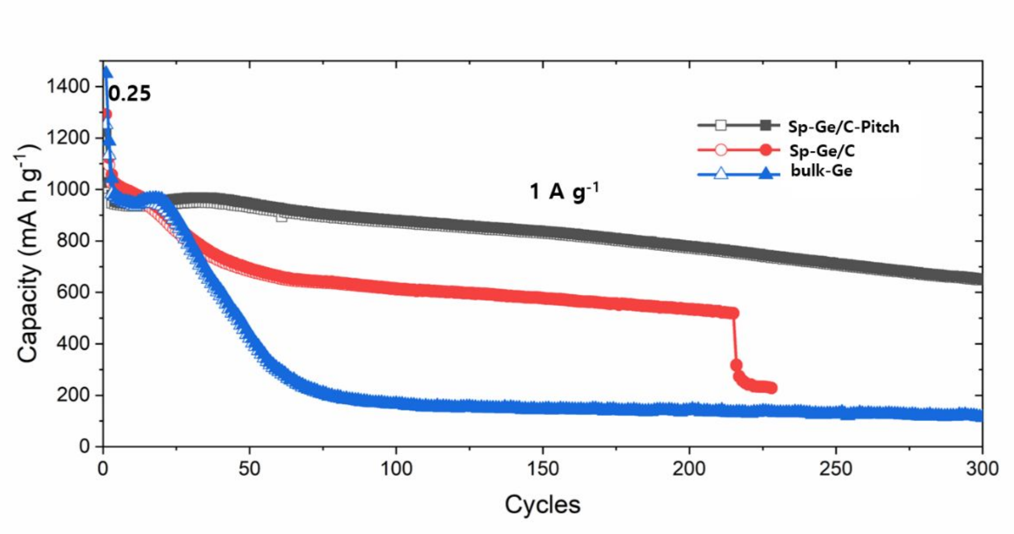

Figure S8. Cycling performance of Sp-Ge/C-Pitch, Sp-Ge/C, and bulk-Ge electrodes for 300 cycles under 0.25 A g^{-1} (2 cycles) and 1 A g^{-1} current densities.

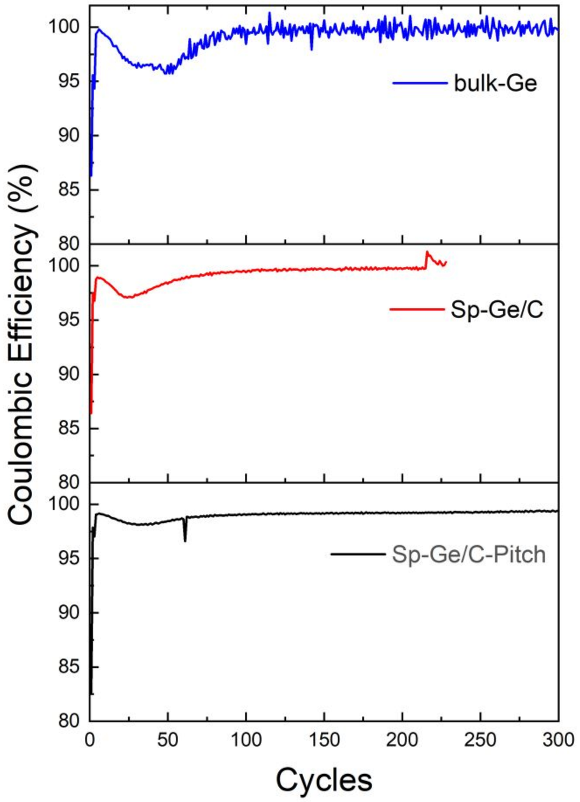

Figure S9. Coulombic efficiency versus cycle plot for Sp-Ge/C-Pitch, Sp-Ge/C, and bulk-Ge electrodes, obtained at 1 A g^{-1} current density.

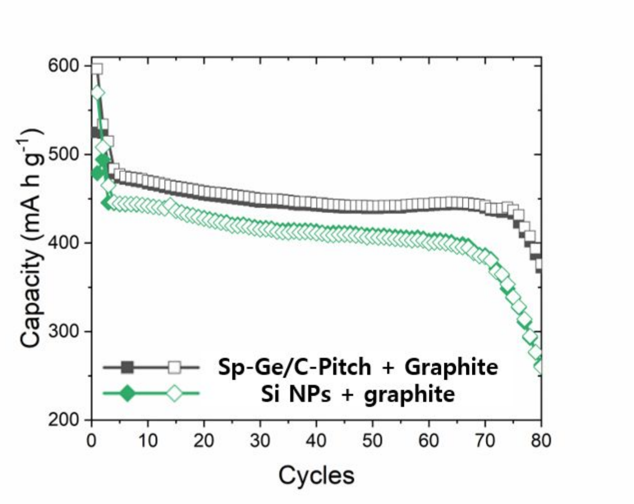

Figure S10. Cycling performance of Sp-Ge/C-Pitch + graphite, Si NPs + graphite electrodes for 80 cycles under 0.05 A g^{-1} (2 cycles) and 0.2 A g^{-1} current densities. Steep capacity decreases in both electrodes around 68~75th cycles is typically due to the unstable cycle performance of lithium metal anodes under high capacity loading (~3.0 mAh cm^{-2}) condition.

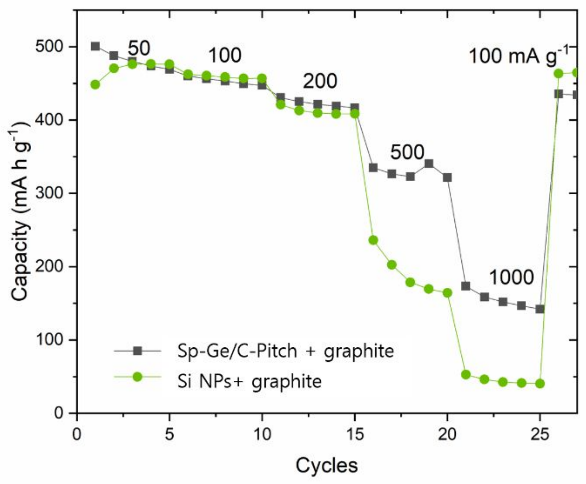

Figure S11. Rate performance test of Sp-Ge-Pitch + graphite electrode and Si NPs + graphite electrode. Capacities of both electrodes were adjusted to same loading level (~500 mA h g^{-1}, ~1.6 g cc^{-1}).

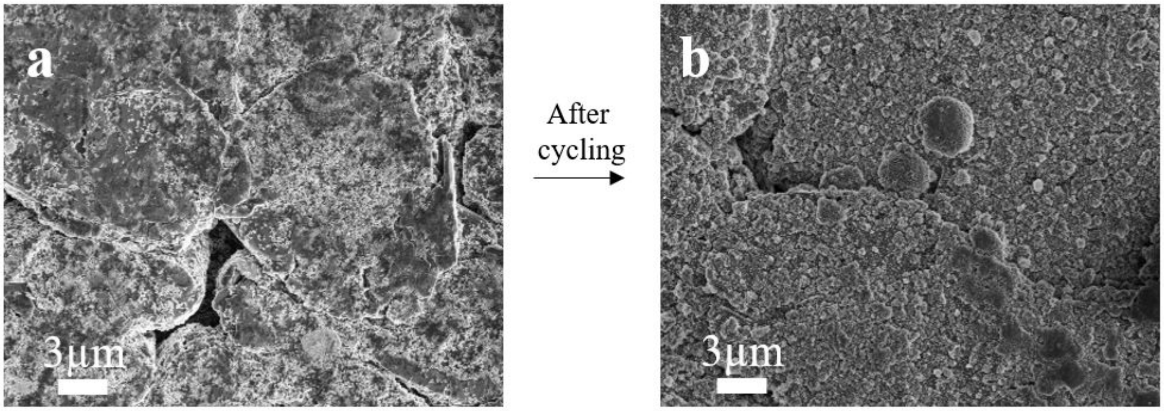

Figure S12. Scanning electron microscope images of Si NPs + graphite electrode (a) before and (b) after cycle test.

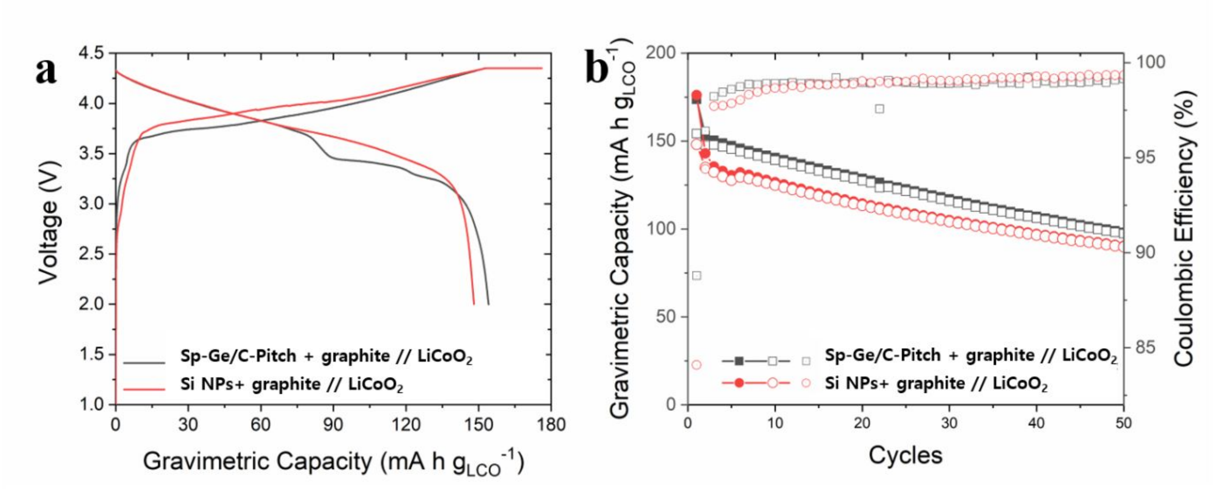

Figure S13. (a) Charge-discharge profiles at formation cycle under 0.1 C-rate of $LiCoO_2$ (LCO, ~16 mA g^{-1}) and (b) cycling performance of Sp-Ge/C-Pitch + graphite // LCO full cell and Si NPs + graphite // LCO full cell under 0.5 C-rate. During charge process, constant voltage was applied at 4.35 V until current decreases to a tenth of applied current.

Supplementary Note. Table S1 and S2 are comparing the performance of Sp-Ge/C Pitch to other reported Ge or Si electrodes in LIB papers. In order to compare the performance as fairly as possible, the representative sample was selected to have best performance in each paper. Since some parameters were not clarified in the referred papers, the data that can be speculated from the graph were entered by our judgement, which is denoted with tilde(~) before the value.

Table S1. Ge-based materials' electrode performance comparison (counter electrode : Li-metal) of Sp-Ge/C-Pitch in this work and other reported modified Ge/C composites.

Material	Cycles [n]	Current density [mA g^{-1}]	Capacity retention [%]	Capacity after n cycles [mAh g^{-1}]	Loading mass [mg/cm^2]	Electrolyte composition[a)]	Ref.
Sp-Ge/C-Pitch	100	1000	91.8		1	1.3M $LiPF_6$ in EC/DEC + 10wt% FEC	This work
Ge/C	50	160	89.5	1095	1	1M $LiPF_6$ in EC/DMC (1:1 by vol%)	1
Ge@C HCS	100	640	90.9	~1000	0.9	1.15M $LiPF_6$ in EC/DMC/DEC (3:4:3 by vol%)	2
C-Ge/C	120	800	74	896	1	1.15M $LiPF_6$ in EC/DMC/DEC (3:4:3 by vol%)	3
Ge@C/RGO	50	50	89.5	940	N/A	1M $LiPF_6$ in EC/DMC/DEC (1:1:1 in wt%) + 2wt% VC	4
Ge@CC	500	800	91.4	1065.2	0.8	1.15M $LiPF_6$ in EC/DMC/DEC (3:4:3 by vol%) + 5wt% FEC	5
3D-Ge/C	1000	3200	88.6	1216	0.5	1M $LiPF_6$ in EC/DMC (1:1 by vol%) + 3wt% FEC	6
C-Ge nanowire	500	320	89	1225	0.1	1M $LiPF_6$ in EC/DMC (1:1 by vol%) + 3wt% VC	7
Ge-NMCF	500	500	80.1	600.9	0.8	1M $LiPF_6$ in EC/DMC (1:1 by vol%)	8

[a)]EC: ethylene carbonate, DEC: diethyl carbonate, DMC: dimethyl carbonate, FEC: fluoroethylene carbonate, VC: vinylene carbonate

Table S2. Performance comparison of graphite-blended electrode in this work and other reported blended electrode. Since there are few papers reporting Ge/graphite blending electrode, Si/graphite blending electrode has been considered either.

Material	G ratio[a)]	Cycles [n]	Current density [mA g^{-1}]	Capacity retention [%]	ICE [%]	Loading mass [mg/cm^2]	Electrode density [g/cc]	Electrolyte composition	Counter electrode	Ref.
Sp-Ge/C-Pitch + G	1:4	60	200	91.2	87.9	6	1.67	1.3M $LiPF_6$ in EC/DEC + 10wt% FEC	Li-metal	This work
Si/Ge/G @C	1:1	100	200	N/A	79.3	1.5	1.15	1M $LiPF_6$ in EC/DMC (1:1 by vol%)	Li-metal	9
Si/C-G	1:2	450	100	82	63	3.9	1.2	1M $LiPF_6$ in EC/DEC (1:1 in wt%) + 10wt% FEC	Li-metal	10
B-Si/CNT@G	1:9	100	100	83.4	~80	11.2	N/A	1M $LiPF_6$ in EC/DEC (1:1 by vol%) + 10wt% FEC	Li-metal	11
Si/C-G	1:2~1:3	300	0.5mA cm^{-2} (charge) 0.75mA cm^{-2} (discharge)	84	95	N/A	N/A	1M $LiPF_6$ in EC/DEC (1:1 in wt%) + 10wt% FEC	NMC111	10
B-Si/CNT@G	1:9	300	180	82.5	96	12	N/A	1M $LiPF_6$ in EC/DEC (1:1 by vol%) + 10wt% FEC	Al2-FCG76 (NCM)	11
GeGrNPs-30h	1:1	200	98.6	84	75	1.2~1.4	N/A	1M $LiPF_6$ in EC/DEC (1:1 by vol%)	Li-metal	12

[a)]Ratio of active material to graphite(G)

References

1. Kim, S.-W.; Ngo, D. T.; Heo, J.; Park, C.-N.; Park, C.-J. Electrodeposited Germanium/Carbon Composite as an Anode Material for Lithium Ion Batteries. *Electrochim. Acta* **2017,** *238*, 319-329.

2. Li, D.; Feng, C.; Liu, H. k.; Guo, Z. Hollow Carbon Spheres with Encapsulated Germanium as an Anode Material for Lithium Ion Batteries. *J. Mater. Chem. A* **2015,** *3* (3), 978-981.

3. Seng, K. H.; Park, M. H.; Guo, Z. P.; Liu, H. K.; Cho, J. Self-Assembled Germanium/Carbon Nanostructures as High-Power Anode Material for the Lithium-Ion Battery. *Angew. Chem.* **2012,** *124* (23), 5755-5759.

4. Xue, D.-J.; Xin, S.; Yan, Y.; Jiang, K.-C.; Yin, Y.-X.; Guo, Y.-G.; Wan, L.-J. Improving the Electrode Performance of Ge through Ge@C Core–Shell Nanoparticles and Graphene Networks. *J. Am. Chem. Soc.* **2012,** *134* (5), 2512-2515.

5. Li, D.; Wang, H.; Liu, H. K.; Guo, Z. A New Strategy for Achieving a High Performance Anode for Lithium Ion Batteries—Encapsulating Germanium Nanoparticles in Carbon Nanoboxes. *Adv. Energy Mater.* **2016,** *6* (5), 1501666.

6. Ngo, D. T.; Le, H. T. T.; Kim, C.; Lee, J.-Y.; Fisher, J. G.; Kim, I.-D.; Park, C.-J. Mass-Scalable Synthesis of 3d Porous Germanium–Carbon Composite Particles as an Ultra-High Rate Anode for Lithium Ion Batteries. *Energy Environ. Sci.* **2015,** *8* (12), 3577-3588.

7. Garcia, A.; Biswas, S.; McNulty, D.; Roy, A.; Raha, S.; Trabesinger, S.; Nicolosi, V.; Singha, A.; Holmes, J. D. One-Step Grown Carbonaceous Germanium Nanowires and Their Application as Highly Efficient Lithium-Ion Battery Anodes. *ACS Appl. Energy Mater.* **2022,** *5* (2), 1922-1932.

8. Liu, J.; Muhammad, S.; Wei, Z.; Zhu, J.; Duan, X. Hierarchical N-Doping Germanium/Carbon Nanofibers as Anode for High-Performance Lithium-Ion and Sodium-Ion Batteries. *Nanotechnology* **2020,** *31* (1), 015402.

9. Chang, L.; Lin, Y.; Wang, K.; Yan, R.; Chen, W.; Zhao, Z.; Yang, Y.; Huang, G.; Chen, W.; Huang, J.; Song, Y. Facile Synthesis of Si/Ge/Graphite@C Composite with Improved Tap Density and Electrochemical Performance. *RSC Adv.* **2023,** *13* (1), 440-447.

10. Li, X.; Yan, P.; Xiao, X.; Woo, J. H.; Wang, C.; Liu, J.; Zhang, J.-G. Design of Porous Si/C–Graphite Electrodes with Long Cycle Stability and Controlled Swelling. *Energy Environ. Sci.* **2017,** *10* (6), 1427-1434.

11. Li, P.; Hwang, J.-Y.; Sun, Y.-K. Nano/Microstructured Silicon–Graphite Composite Anode for High-Energy-Density Li-Ion Battery. *ACS Nano* **2019,** *13* (2), 2624-2633.

12. Sultana, I.; Rahman, M. M.; Glushenkov, A. M.; Mateti, S.; Tanwar, K.; Huang, S.; Chen, Y. Nano Germanium Incorporated Thin Graphite Nanoplatelets: A Novel Germanium Based Lithium-Ion Battery Anode with Enhanced Electrochemical Performance. *Electrochim. Acta* **2021,** *391*, 139001.
